# Sequence-Dependent Fluorescence of Cyanine Dyes on Microarrays

**DOI:** 10.1371/journal.pone.0022177

**Published:** 2011-07-25

**Authors:** Christy Agbavwe, Mark M. Somoza

**Affiliations:** Institute of Inorganic Chemistry, University of Vienna, Vienna, Austria; University of North Carolina at Charlotte, United States of America

## Abstract

Cy3 and Cy5 are among the most commonly used oligonucleotide labeling molecules. Studies of nucleic acid structure and dynamics use these dyes, and they are ubiquitous in microarray experiments. They are sensitive to their environment and have higher quantum yield when bound to DNA. The fluorescent intensity of terminal cyanine dyes is also known to be significantly dependent on the base sequence of the oligonucleotide. We have developed a very precise and high-throughput method to evaluate the sequence dependence of oligonucleotide labeling dyes using microarrays and have applied the method to Cy3 and Cy5. We used light-directed *in-situ* synthesis of terminally-labeled microarrays to determine the fluorescence intensity of each dye on all 1024 possible 5′-labeled 5-mers. Their intensity is sensitive to all five bases. Their fluorescence is higher with 5′ guanines, and adenines in subsequent positions. Cytosine suppresses fluorescence. Intensity falls by half over the range of all 5-mers for Cy3, and two-thirds for Cy5. Labeling with 5′-biotin-streptavidin-Cy3/-Cy5 gives a completely different sequence dependence and greatly reduces fluorescence compared with direct terminal labeling.

## Introduction

When Cy3, Cy5 or other dyes are used as oligonucleotide labels in, e.g., microarray experiments, fluorescent *in situ* hybridization (FISH), real-time PCR (RT-PCR), and FRET studies, sequence-dependent fluorescence is often assumed not to significantly affect the results. Although significant concerns about reproducibility and biases in microarray experiments focus primarily on experimental design, data analysis and interplatform reproducibility [1], [2], [3], gene-specific dye biases have also been extensively discussed [4], [5], [6], [7], [8], [9], and even traced to the nucleobase proportion in the mRNA and oligonucleotide probes [10]. Dye bias is not widely discussed for other biotechnology methodologies based on oligonucleotide labeling, even though similar biases should apply.

Fluorescent molecules are generally sensitive to environmental conditions, and their response to properties such as viscosity [11], polarizability [12], pH [13], elasticity [14] and polarity [15] can be used to elucidate biophysical properties of proteins, nucleic acids, and cellular membranes and compartments. The cyanine dyes are not considered particularly environmentally sensitive [16], although the Cy3/Cy5 intensity ratio has been used to measure cytoplasm viscosity [11]. The Cy3/Cy5 fluorescence intensity ratio is related to viscosity because the three-carbon polymethine chain connecting the two indole rings in Cy3 is relatively flexible, and the molecule can undergo a cis-trans isomerization from the first excited singlet state which competes with fluorescence [17], [18], [19], [20]. In more viscous environments, isomerization is restricted, and more of the energy from absorbed photons is released as fluorescence. Cy5 has a five-carbon chain connecting the rings, but assumes a more rigid and stable planar configuration which favors the florescence pathway to the ground state [11]. Cy5 has a isomerization rate three times lower than that of Cy3 [18]. Tethering to DNA should restrict the range of motions available to these dyes, and indeed, NMR studies have shown that both Cy3 and Cy5 are positioned in a capping configuration in the way of an additional base pair at the end of the double helix [21], [22], increasing fluorescence quantum yield and lifetime [20]. Time-resolved fluorescence anisotropy measurements of Cy3 bound to the 5′ end of DNA have shown that some of the anisotropy decay can be attributed to motion of the dye relative to the DNA, indicating that the motions of the dye molecules are not fully constrained [20]. The same study also showed a greater degree of freedom for Cy3 bound to double-stranded DNA vs. single-stranded DNA, and a concomitantly lower isomerization, and higher fluorescence quantum yield and lifetime for Cy3-ssDNA. Even without tethering, Cy3 binds to nucleobase monophosphates [23], and with stronger affinity to the purines, possibly driven by π-stacking interactions. In both of these cases, the binding leads to an enhancement of fluorescence following the nucleobase order: dG>dA≫dT≈dC>buffer only.

Recent experiments have shown that Cy3 fluorescence is surprisingly sensitive to not just the closest base, but to several additional bases farther away [24]. These results were based on 20 Cy3 5′-end-labeled ssDNA homo- and mixed-sequence oligonucleotides, as well as five sequences with an abasic site, and indicated a factor of two intensity difference between the most and least fluorescent sequences. As with other experiments on cyanine dye/DNA interactions, the results were attributed to changes in the photo-isomerization rate, with sequence-dependent variations due to chain flexibility and formation of secondary structures.

Because quantifying the sequence-dependent fluorescence of DNA labeling molecules is important for accurate interpretation of experimental data derived from labeled DNA, we have developed a very precise and high-throughput method to evaluate this sequence sensitivity of dyes using microarrays and have applied the method to Cy3 and Cy5. In this study, we determined the detailed sequence-dependence of both Cy3 and Cy5 fluorescence intensity by synthesizing all 1024 possible DNA 5′dye-labeled 5-mers on DNA microarrays. We also explored the possibility of isolating the dyes from sequence effects by terminating the DNA sequences with a biotin phosphoramidite and attaching the dyes via Cy3- and Cy5-labeled streptavidin. The microarrays were synthesized using maskless array synthesis (MAS), a fabrication method developed for *in situ* synthesis of high-density DNA microarrays for genomics applications [25]. Because the synthesis environment is highly uniform across the array surface and many replicates of each sequence can be included on the same microarray, the sequence-dependent relative fluorescence of thousands of sequences can be accurately determined in a single experiment.

## Methods

### Microarray synthesis

The microarray substrates were prepared from Arrayit Superclean Slides functionalized with N-(3-triethoxysilylpropyl)-4-hydroxybutryamide (Gelest SIT8189.5). The slides were loaded in a metal rack and completely covered with a 550 ml of a solution consisting of 10 mg of the silane in 95∶5 (v/v) ethanol:water and 1 ml acetic acid. The slides were covered and gently agitated for 30 min and then rinsed twice in pure ethanol, with gentle agitation, for 15 min. The slides were then drained and cured overnight in a preheated vacuum oven (120 C). The vacuum oven heat was turned off after the vacuum was established. The slides were stored in a desiccator cabinet.

Cy3, Cy5 and biotin terminated oligonucleotides were synthesized on microarrays using the technique of maskless array synthesis (MAS), a proven fabrication method developed for *in situ* synthesis of high-density DNA microarrays for genomics applications [25]. The MAS instrument can be conceptually divided into two components, an optical system and a chemical delivery system. The chemical side consists of a modified Perspective Biosystems Expedite 8909 synthesizer, which is used to deliver solvents and reagents to the functionalized glass surface where the microarray synthesis takes place. The optical system is similar to that of a photolithographic system, but it uses a digital micromirror device (DMD) in place of photomasks to pattern the ultraviolet light from a mercury lamp. The pattern displayed on the micromirror device is transferred to the synthesis surface, where the array layout and oligonucleotide sequences are determined by selective removal of the photocleavable protecting groups on the phosphoramidites at the 5′ termini of the oligonucleotides.

Reagent delivery and light exposures are synchronized and controlled by a computer, which also stores and orders the display of virtual masks on the micromirror array. The chemistry is similar to that used in conventional solid-phase oligonucleotide synthesis. The primary modification is the use of phosphoramidites with a 5′-nitrophenylpropyloxycarbonyl (NPPOC) photocleavable protecting group. Upon absorption of a photon near 365 nm, the NPPOC group drops off, leaving a 5′-hydroxyl terminus which is able to react with an activated phosphoramidite during the next synthetic cycle.

During ultraviolet exposure, NPPOC removal is promoted with an exposure solvent consisting of 1% (m/v) imidazole in dimethyl sulfoxide (DMSO). Capping with a 1∶1 mix of tertbutylphenoxyacetyl acetic anhydride in tetrahydrofuran (Cap A) and 10% N-methylimidazole in tetrahydrofuran/pyridine (8∶1) (Cap B) was used to ensure the sequence fidelity of the labeled sequences. Representative chemical synthesis protocols are given in Table S1. Following each synthesis, the glass substrate bearing the microarray was washed vigorously with acetonitrile for two hours to remove most of the remaining uncoupled Cy3 or Cy5 phosphoramidite, which tend to stick non-specifically to the glass surface and to the array oligonucleotides. The base and phosphate protecting groups were removed by immersing the glass slide into 1∶1 (v/v) ethylenediamine in ethanol for two hours at room temperature. Following deprotection, the microarrays were washed twice with distilled water or acetonitrile and dried with argon. The NPPOC phosphoramidites and all the synthesis reagents were purchased from Sigma-Aldrich; the Cy3 phosphoramidite from GE Life Sciences; and Cy5 and biotin phosphoramidites from Glen Research. Microarrays synthesized with 5′-biotin were incubated with either streptavidin-Cy3 or streptavidin-Cy5 (ZyMax Grade from Invitrogen).

### Microarray design

With the experimental aim of determining the sequence dependence of cyanine dye fluorescence, the set of all 1024 possible single-stranded DNA 5-mers was chosen as the optimal set for inclusion on each microarray. The resolution of the digital micromirror device, 768×1024, allows for a maximum about 750 thousand experimental sequences per array, after allowing for quality control and alignment sequences. Nevertheless, we know from experience that the signal-to-noise level in terminal labeling experiments with fluorescent dyes is significantly worse than with the typical hybridization experiments carried out on microarrays with dye-labeled complementary sequences, and therefore larger microarray areas and more replicates per sequence are helpful. The 1024 sequences were laid out in a 25 in 36 pattern, that is, each “feature” (contiguous area were a single sequence is synthesized) on the microarray corresponded to a 5 by 5 block of mirrors surrounded by a one-mirror-sized margin where no DNA was synthesized. Each of the 1024 single-sequence features was replicated 20 or 21 times on each microarray.

### Sequence design

An important consideration with *in situ* microarray synthesis is the coupling efficiency of each of the four DNA nucleotide phosphoramidites. The purine phosphoramidites typically couple worse than the pyrimidines, which would lead to differential fluorescence of terminally labeled oligonucleotides containing different proportions of bases, even before accounting for the expected sequence-dependent modulation of fluorescence of the cyanine dyes. In principle, it might be possible to correct the sequence-dependent fluorescence intensity data by incorporating a correction factor based on the coupling yield of each base in the sequence, however the coupling efficiencies of the phosphoramidites used in microarray synthesis are measured with fluorescent dye terminal labeling experiments [26], [27], [28], [29], and are thus likely inaccurate based on the results presented herein. To eliminate coupling yield differences between sequences, the sequences synthesized on the microarray had the following design:
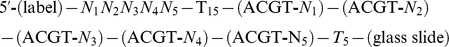
The *N*_i_ represent the 5-mer experimental sequences. These are separated by a 15-thymidine linker from a 15-mer with bases customized as shown, with each of the experimental bases subtracted from five copies of sets of all four DNA bases. This design, in conjunction with capping following each coupling, ensures that all of the sequences which receive the 5′-dye will have the same number of each base and hence equal number density on the microarray surface.

There are two possible ways by which the terminal label may interact with DNA bases: through-the-stack interactions to bases close to the 5′ end, and dye intercalation with downstream bases enabled by the very high flexibility of ssDNA (the persistence length is ∼2 nm or the length of ∼3 nucleotides [30]). The 15-thymidine linker was hypothesized to be sufficiently long to prevent propagation of through-the-stack interactions from the downstream bases used to ensure the equal number density of each labeled sequence on the microarray. Although there is no evidence in the literature that Cy3/5 can intercalate ssDNA, the high proportion of thymidines also maximizes the flexibility of the sequences, which ensures that if the dyes can associate with downstream bases, they have an almost equal opportunity to associate with all bases. Since all of the sequences on the microarray have the same proportion of each base, this leads to sequence-dependent fluorescence data that is dominated by through-the-stack interactions between the dyes and the 5-mer experimental sequence.

### Data extraction and analysis

The microarrays were scanned with a GenePix 4100A at 5 µm resolution and with detector voltages set to give similar intensity ranges for both Cy3 and Cy5, and no saturated pixels, 350 and 450 volts, respectively. The fluorescence intensity data was extracted from the scan image with NimbleScan v2.1 software from Roche-NimbleGen and further processed in Excel. For each microarray, fluorescence intensity values were calculated as the average of the 20 or 21 replicates of each sequence, which were randomly located on each microarray. Error was calculated as the standard error of the mean and, for most sequences, is in the range of 1% to 2% of the maximum intensity for the Cy3-labeled sequences, and 2% to 4% of the maximum intensity for the Cy5-labeled sequences. The consensus sequence figures for each of the four labeling methods were generated by ranking the 1024 sequences by fluorescence intensity and then dividing the sequences into eight bins spanning equal ranges of intensity. Consensus logos for the sequences in each of these octiles of fluorescence intensity were generated using Weblogo (http://weblogo.berkeley.edu/) [31]. Each of the eight consensus sequence logos per fluorescent label represents one eighth of the intensity range and are arranged together left to right in order of decreasing intensity to compactly depict the relationship between sequence and fluorescence for the entire dataset. The error bars in the sequence logos are determined from the number of sequences in each octile of intensity.

## Results

**Figure 1:**
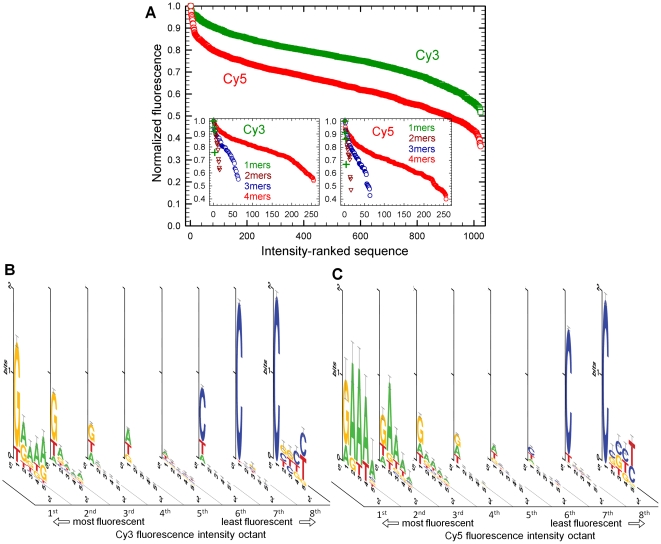
Oligonucleotide labeling with Cy3 and Cy5 phosphoramidites. (A) Fluorescence intensity of Cy3 and Cy5 end-labeled 5-mers, ranked from most to least intense. The Cy3 curve drops by about half of the maximum intensity, while the Cy5 curve drops by about two-thirds. The shape of the curves suggests that the intensity range for both dyes will increase with additional sequence length. However the insets, which each plot the four *N*_x_T_5-x_ subsets of the same data, preserve the inverse sigmoidal shape, and the trend can be extrapolated to indicate a small additional range for Cy3. Fluorescence intensity consensus sequences of all 1024 DNA 5-mers 5′-end-labeled using (B) Cy3 and (C) Cy5, synthesized simultaneously on a microarray. The fluorescent range was equally divided into eight bins of equal intensity range, and the consensus sequence for all the 5-mers in each bin is plotted for each octile.

Both Cy3 and Cy5 fluorescence show strong sequence dependence. The sequence pattern leading to both high and low intensity is well-defined and similar for both labels. The change in intensity of Cy3 and Cy5 over the range of all possible 5-mer sequences is large, as shown in Figure 1a. Cy3 fluorescence drops by almost half over the range of sequences, while the intensity from Cy5 drops by almost two-thirds. The consensus sequences for the most and least Cy3 and Cy5 fluorescent sequences show that longer sequences would extend the fluorescent range to both higher and lower values. For example, the first-ranked Cy3 sequence, GAAAA, leads to about 5% higher fluorescence than the 27th ranked sequence GAAAT, so adding one or more additional dAs after the dG would likely improve Cy3 fluorescence. Similarly, allowing sequences with more than five dCs should decrease relative fluorescence at the other end. The upward and downward turns in the inverse sigmoid curves in Figure 1a suggest that the four-fold increase in the number of sequences added with a sixth base would greatly extend the range between the brightest and the darkest labeled sequence. Similar plots for subsets of the data of the form *N*_1_ *N*_2_ *N*_3_ *N*_4_T through *N*_1_TTTTT are shown in Figure 1a-inset. The minimum intensity values for each of these data subsets follow a single exponential decline to a minimum relative fluorescence of 51%, indicating that the labeled 5-mers account for about 97% of the possible range in fluorescence for Cy3 (Figure 1a-inset). Applying this analysis to the Cy5 data, there is no clear exponential convergence to a minimum, suggesting considerable additional range of fluorescence for longer sequences.

Cy3 is most intense when immediately adjacent to a dG and, to a lesser extent, a dT. Additional dGs in more distal positions of the 5-mer also promote fluorescence, but the greatest intensity results from dAs following the initial dG. If the sequences are divided into octiles of fluorescence intensity (Figure 1b), the most intense octile contains only sequences which begin with dG and are followed by several dAs or dGs, or sequences which start with dT and are followed only by a mix of dTs and dAs (see Figure S1 and Figure S2 for the full data set; see Data S1 for the full dataset in computer-readable format). In the second, third, and fourth Cy3 fluorescence intensity octiles, the same base pattern continues. However, the importance of dG in the first position gradually declines, and dA appears in this position; the role of specific bases in positions two through five also lose importance. Cy3 fluorescence is low when immediately followed by dC; all but one of the sequences in the lowest quartile of fluorescence have a dC in this position. dAs rarely appear in the lowest octile of fluorescence, and then only in sequences with a minimum of three dCs.

The sequence-dependent pattern of Cy5 fluorescence intensity is very similar to that of Cy3, but Cy5 is more sensitive to the distal bases. Although Cy5 fluorescence is also strongly favored by a dG in the first position followed by multiple dAs, a dA in the first position is also highly favorable (Figure 1c). dA in distal positions is more important than an initial dG, and the amplitude of this effect persists in the 2nd and 3rd intensity octiles. On the low fluorescence side, Cy3- and Cy5-labeled sequences show an almost identical pattern: dC is strongly favored, particularly in the first position, and dA is almost absent. Table 1 gives the ten sequences resulting in the highest and lowest fluorescence of Cy3 and Cy5. The complete Cy5 dataset is shown in Figure S3 and Figure S4.

**Table 1:** Normalized relative fluorescence intensity of the ten brightest and darkest 5-mers 5′-labeled with Cy3 or Cy5 phosphoramidites.

Rank	Cy3-sequence	Normalized fluorescence†	Cy5-Sequence	Normalized fluorescence†
1	GAAAA	1.000±0.015	GATAA	1.000±0.032
2	TATAA	0.999±0.013	GGAAA	1.000±0.048
3	GGGAA	0.989±0.014	GAAAG	0.974±0.030
4	GGAAG	0.986±0.015	GAAAA	0.959±0.027
5	GAAAG	0.976±0.017	GAATA	0.955±0.031
6	GAATG	0.975±0.016	AAATA	0.947±0.040
7	GGAGG	0.968±0.016	AAAAA	0.941±0.032
8	GGAGA	0.966±0.016	GAAAC	0.933±0.033
9	GACAA	0.963±0.018	GAAAT	0.920±0.026
10	GGGGA	0.962±0.017	AAAAG	0.917±0.033
1015	CCTCC	0.542±0.017	CCCCA	0.408±0.014
1016	CGTCT	0.542±0.013	CGGCT	0.406±0.027
1017	CCTCT	0.541±0.017	CCCCT	0.402±0.024
1018	CCTTT	0.539±0.015	CGGCG	0.396±0.015
1019	CCGCC	0.535±0.018	CGGGT	0.394±0.035
1020	CGTTC	0.535±0.011	CGCCC	0.390±0.023
1021	CCTTC	0.532±0.015	CTGCC	0.377±0.011
1022	CGTTT	0.526±0.018	CGTTC	0.374±0.009
1023	CCCCC	0.519±0.016	CGTTT	0.368±0.018
1024	CGGTT	0.516±0.017	CGGTC	0.362±0.019

† Error calculated as standard error of the mean.

**Figure 2:**
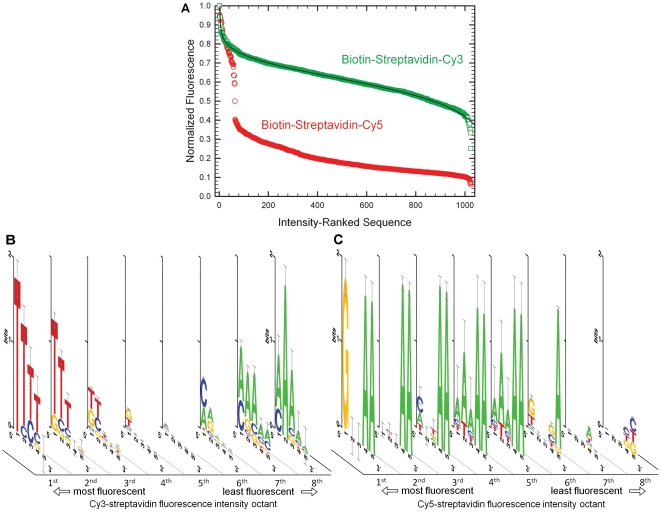
Oligonucleotide labeling with biotin-streptavidin-Cy3 and -Cy5. (A) Fluorescence intensity of Cy3 and Cy5 end-labeled 5-mers, ranked from most to least intense. The fluorescence intensity of the Cy3-streptavidin-biotin conjugate drops by about 75% of maximum intensity over the range of sequences; in the case of Cy5, the intensity drops by about 95%. Fluorescence intensity consensus sequences of all 1024 DNA 5-mers with 5′-biotin and labeled using (B) streptavidin-Cy3 conjugation and (C) streptavidin-Cy5 conjugation. The fluorescent range was equally divided into eight bins of equal intensity range, and the consensus sequence for all the 5-mers in each bin is plotted for each octile.

DNA labeling via biotin-streptavidin affinity is a common practice and we hypothesized that while the fluorescence of the dyes might be strongly perturbed by interactions with the protein, there would be less coupling to the bases and a weaker sequence dependence of fluorescence. However, the magnitude of the sequence-dependent fluorescence intensity shift is much larger for labeling via the biotin-streptavidin bridge versus direct terminal labeling. Figure 2a shows that Cy3 intensity falls by ∼75% of its peak value, and Cy5 intensity can be quenched almost completely by some sequences. The shapes of the curves for Cy3 and Cy5 in Figure 2a are also inverse sigmoidals, but highly distorted for Cy5 due to the strong influence of the 64 3′-dA_2_ 5-mers.

Figure 2b,c show the consensus sequences for the fluorescence intensity octiles of Cy3 and Cy5. The biotin-streptavidin bridge results in a strong shift in the consensus for Cy3 sequences, with dT strongly favoring fluorescence and dA quenching fluorescence. dC appears often in the first position of weakly fluorescent sequences but both dG and dC are less influential than in the case of direct attachment of the dye to oligonucleotides (Figure S5 and Figure S6). The base pattern for biotin-streptavidin-Cy5 sequences is completely different, with dA required in positions 4 and 5 for strong fluorescence. All of the 64 possible 5-mers ending with dA_2_ are in the 64 most fluorescent sequences. Once the possible dA_2_ sequences are exhausted, singly dA terminated sequences follow in importance. The ten most fluorescent sequences also have a dG in the first position (Figure S7 and Figure S8), but otherwise this base, along with dC, is mostly absent from the consensus sequences. A shift in the nucleoside pattern is not unexpected since the dyes are not longer able to form direct stacking interactions with the DNA. In spite of the protein intermediate, it is clear that the Cy dyes are interacting strongly with the 5-mer experimental sequence (and not e.g., with other nucleosides further down via a bend in the chain) as evidenced by the well-defined pattern seen in the consensus sequences.

## Discussion

### Direct labeling with Cy3 and Cy5 phosphoramidites

The results from the microarray experiments provide a clear and high resolution pattern of the sequence dependence of Cy3 and Cy5 fluorescence. The results are generally consistent with previous experiments on the fluorescence of cyanine dyes interacting with ssDNA. Mikelsons et al. [32] showed that an intercalating unsymmetrical monomethine cyanine dye derived from thiazole orange associates strongly with poly(dG) and poly(dA) but not with poly(dT) and poly(dC), resulting in relative fluorescence intensities following the pattern: dG>dA≫dC>dT> no DNA (100, 39, 2.3, 1.8 and 0.5, respectively). Their computer simulations indicated that the dyes associate poorly with poly(dC) and poly(dT), while binding strongly to poly(dG) and poly(dA). Harvey et al. [23] studied the interactions of Cy3 with nucleoside monophosphates and also found a similar pattern of nucleobase-specific enhancement of fluorescence, dG>dA>dT>dC>no DNA, but a much narrower range of intensity differences, about a factor of two between dG and dC, and with dC only modestly promoting Cy3 fluorescence over the buffer alone. All of these results are consistent with a model in which π-π interactions between the dye and the nucleobase decrease the rate of fluorescence quenching by photoisomerization, since the purines have a more extensive π-system.

When cyanine dyes are covalently attached to the 5′-end of oligonucleotides, there is the potential for stronger interactions between the dye and the sequence of nucleotides. This arrangement is also highly relevant since cyanine dyes are typically used as 5′-fluorescent probes in many molecular biology, biotechnology and biophysics applications. Harvey et al. were the first to measure the fluorescence efficiency and mean lifetimes of 5′-Cy3 end-labeled ssDNA [24]. They examined homo- and mixed-sequence oligonucleotides, as well five sequences with an abasic site, and found more than a factor of two intensity difference between the most and least fluorescent sequences. They also found that the intensity of Cy3 was sensitive to single base or sequence differences beyond the sixth nucleotide. Their data is, however, not fully consistent with the intensity trends from the nucleoside monophosphate and homopolymer data, for example, the Cy3-poly(dA) sequence has the lowest fluorescence of their experimental sequences, and several sequences with a 5′-dC are among the most fluorescent. Also, because the 20 sequences are a very small subset of all the possible multibase oligonucleotides, the pattern in the sequence-dependent intensity data could not be fully established, nor could the full magnitude of sequence-dependent fluorescence variability be determined. The extensive datasets herein substantially determine both the relevant sequence patterns and the magnitude of the fluorescence variability, for both Cy3 and Cy5, and will allow detailed evaluations of dye biases in both single and two color microarray experiments, as well as in other labeling applications.

The results we present here are consistent with the nucleoside monophosphate and homopolymer data, with the intensity of Cy3 following the pattern dG>dA>dT>dC for 5-mer homopolymers. Specifically, among the 1024 possible Cy3-labelled 5-mers ranked from high to low fluorescence, they are in positions 18, 243, 706 and 1023, with relative fluorescence values of 1, 0.87, 0.75 and 0.54, respectively. In the case of Cy5, which was not addressed in the previous studies, the positions of poly(dA) and poly(dG) are reversed, and the homopolymer 5-mer sequences lead to relative fluorescence values of 1, 0.88, 0.61 and 0.46, for dA, dG, dT and dC respectively. Looking at the full dataset leads to similar results. Both Cy3 and Cy5 fluoresce more with a adjacent dG and fluoresce least with an adjacent dC, but the effects of both dA and dT are more complex. It is clear from Figure 1b,c that dA strongly favors fluorescence, but particularly in the second through fifth position, with dA rarely appearing in the first position in the most intense Cy3-labeled sequences. For the most intense Cy5-labeled sequences, dA appears occasionally in the first position, but the second through fifth position are preferred. The effect of dT is more subtle and a departure from what one might expect from the nucleoside monophosphate and homopolymer data. This base appears in both the most and least fluorescence sequences. The second most fluorescent Cy3 sequence is TATAA, which has essentially the same intensity as the first place sequence GAAAA (see Table 1). TATAA is in the 13^th^ position in the Cy5 dataset. Several other sequences starting with dT are also associated with high Cy3 and Cy5 fluorescence and share the common feature of consisting primarily of dT and dA's.

It is well-known that the fluorescence intensity of many conjugated dyes, including fluorescein [33], pyrene [34], coumarin [35] and Rhodamine 6G [36] is dependent on the nucleic acid sequence. In all of these cases, the primary mechanism has been determined to be photoinduced charge transfer between the nucleotides and the dye. The quenching efficiency is base-specific and follows the redox-potential sequence of the four bases, dG<dA<dC<dT, when the bases are reduced, and the reverse order when the bases are oxidized [35]. The electrochemical properties of cyanine dyes in general, and Cy3 and Cy5 in particular, have been determined and indicate that fluorescence quenching by photoinduced charge transfer with nucleobases is not thermodynamically favorable [37], [38].

The mechanism proposed for sequence-dependent changes in intensity of cyanine dyes conjugated with oligonucleotides is the modulation of the potential energy barrier for rotational isomerization in the excited state [17], [18], [19], [20], [23], [24]. While experiments and simulations indicate that cyanine dyes associate closely with nucleobases via π-stacking interactions, and therefore fluoresce more when adjacent to purines, the mechanism by which the rate of rotational isomerization can be affected by more distant bases remains to be determined.

Sequence-dependent rigidity of single-stranded DNA is hypothesized to be an important physical variable in the observed sequence-dependent fluorescence of Cy3 and Cy5. The nucleobase immediately adjacent to the dye directly hinders rotational isomerization, but its ability to do so may be enhanced by it placement in a more rigid sequence of bases. This is analogous to the increase stability observed in short duplexes when an unpaired base is added at one end [39], [40]. Purine stacks in single-stranded DNA are more rigid than pyrimidine stacks, and purine-pyrimidine stacks have an intermediate rigidity [41], [42]. Guckian et al. [43] calculated the stacking area for the nucleobases and arrived at the order: dG (139 Å^2^)>dA (128 Å^2^)>dC (102 Å^2^)>dT (95 Å^2^), based on B-form stacking geometry. They also determined the free energy for stacking (ΔΔG°) and arrived at the order dA≫dG>dT≈dC (2.0, 1.3, 1.1 and 1.0, all ±0.2), based on ssDNA to dsDNA equilibrium experiments. Other experiments have given roughly similar results: dA≈dG>dT>dC, based on dangling tetranucleotides [44], and rA≈rG>rU>rC, based on 3′-end dangling on RNA duplexes [39]. Although dG has a somewhat lower free energy for stacking within DNA relative to dA, it may stack more effectively with the cyanine dyes, based on the results presented here, as well as the homopolymer [32] and nucleoside monophosphate data [23]. Superior dA stacking within the oligonucleotide may then stabilize the dG-dye interaction to explain the strong correlation between strong fluorescence and the consensus sequence GAAAA shown in Figure 1b. The homopolymer and nucleoside monophosphate data also indicate that Cy3 associates poorly with dC; since dC also poorly promotes stacking stability within oligonucleotides, sequences of the form CCCCC should be expected to result in poor fluorescence. This is only partially true based on the least fluorescent consensus sequences for Cy3 in Figure 1b. While the sequence CCCCC is in second to last position, and dC is the most common nucleotide in the least fluorescent sequences, dG appears with similar frequency; dA is almost absent.

For Cy5, there is less data in the literature for comparison, however, Cy3 and Cy5 are very similar and can be expected to interact in similar ways with nucleobases. Although in our homopolymer 5-mer data subset, Cy5, in contrast to Cy3, fluoresces more with poly(dA) than with poly(dG), the consensus sequence for the most fluorescent Cy5-labeled oligonucleotides has a similar pattern as for Cy3, and is thus consistent with the base stacking rigidity hypothesis. The main difference is that dA promotes fluorescence more strongly in all positions (Figure 1c). Poorly fluorescent Cy5-labeled oligonucleotides also have a very similar consensus sequence to that of Cy3. While sequence-dependent rigidity may explain much of the data for Cy3 and Cy5 labeled oligonucleotides, other mechanisms must play a role since both datasets include highly fluorescent sequences such as TATAA and TATAT, which, based on the homopolymer and nucleoside monophosphate data, as well as on the base-stacking data, should lead to poor fluorescence.

The experiments described herein were designed to determine the modulation of fluorescence intensity due to interactions with bases close to the 5′ end attachment point of the dye via short-range stacking interactions. Effects on the relative fluorescence measurements due to longer-range interactions enabled by the flexibility of ssDNA, i.e., stacking or intercalation of the dyes between bases distant from the 5′ end, are largely excluded by the experimental design (at long length scales, all sequence have the same nucleobase composition—see Microarray design section), therefore the present results cannot exclude the possibility that these interactions exist. If intercalation interactions are possible, they would likely further extend the range of sequence-dependent modulation of fluorescence intensity. There are some indications in the data that such interactions do take place. Specifically, in a few of the consensus sequences (Figure 1b,c) there is a noticeable increase in the information content for positions 4 or 5 nucleobases distant from the dye, relative to positions 2 or 3. These last two bases in the experimental sequence are likely the first two bases that could be subject to dye intercalation given the ∼3 nucleotide persistence length of ssDNA.

**Figure 3:**
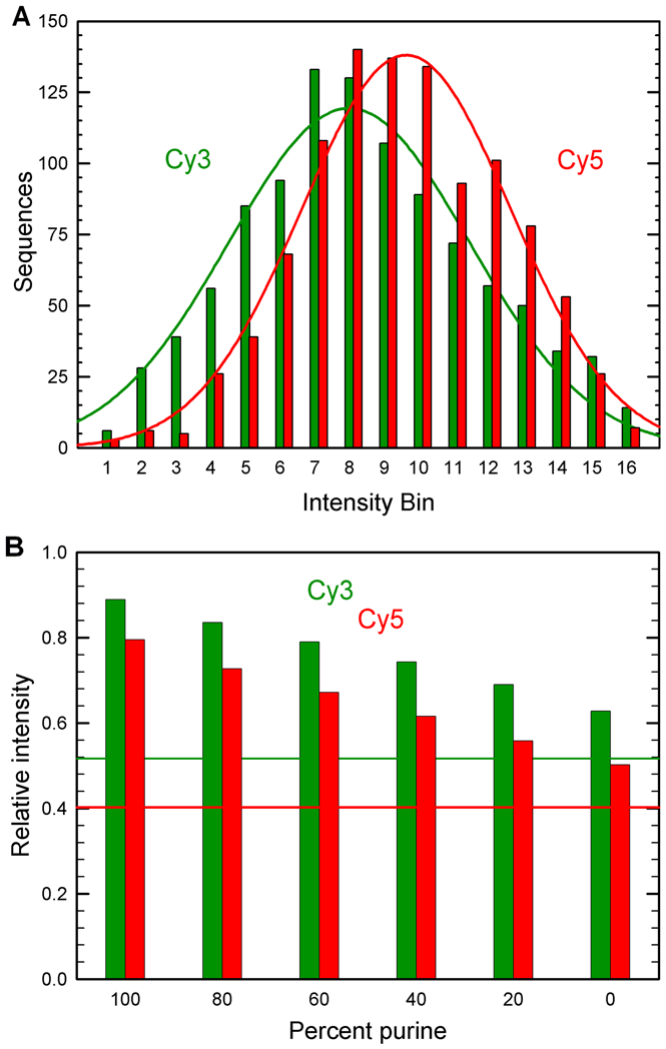
Intensity distribution of oligonucleotides labeled with Cy3 and Cy5 phosphoramidites. (A) Intensity distribution for Cy3 and Cy5 end-labeled 5-mers divided into 16 bins of equal intensity range. The histograms have been fitted with Gaussian functions (Cy3: *A* = 119, *x*_0_ = 8.0, *σ* = 3.5; Cy5: *A* = 138, *x*_0_ = 9.4, *σ* = 3.0). (B) Effect of purine vs. pyrimidine content of 5-mers on the normalized intensity of Cy3- and Cy5-labels. The horizontal lines represent the intensity of the least fluorescent Cy3 and Cy5 end-labeled 5-mers.

The shape of curves in Figure 1a can be interpreted as cumulative distribution functions with normalized relative florescence as the variable, and indicate that the fluorescence intensity of Cy3 and Cy5 on random sequences have probability density functions approximating normal distributions. Figure 3a shows this intensity distribution for the Cy3 and Cy5 data divided into 16 intensity bins and fitted with Gaussian functions (Cy3: *A* = 119, *x*_0_ = 8.0, *σ* = 3.5; Cy5: *A* = 138, *x*_0_ = 9.4, *σ* = 3.0). This interpretation is consistent with the consensus sequences in Figure 1b,c, which show that the relative abundance of purines over pyrimidines promotes fluorescence. Most random 5-mer sequences will contain a mix of purines and pyrimidines, which will result in intermediate fluorescence in the central region of the distribution. Only a few random sequences will contain mostly purines or mostly pyrimidines, resulting in, respectively, fluorescence at the high and low tails of the intensity distribution. Figure 3b shows the relationship between purine vs. pyrimidine content of the sequences and fluorescence intensity. These statistics support the hypothesis that local chain flexibility mediates Cy3 and Cy5 fluorescence, as purines result in higher ssDNA rigidity. Other factors, beyond the purine or pyrimidine content of the sequence, have lesser but still measurable affects on the distribution, including the higher fluorescence associated with a 5′ dG and mixed dT-dA sequences, as discussed previously.

### Biotin-steptavidin-Cy3 and -Cy5 labeled sequences

After establishing that direct labeling of oligonucleotides with Cy3 and Cy5 phosphoramidites leads to significant sequence-dependent range of fluorescence intensity, we hypothesized that ending the same sequences with a biotin phosphoramidite and then labeling with Cy3- or Cy5-conjugated streptavidin would eliminate or greatly reduce the sequence effects. As shown in Figure 2, the result completely contradicted the hypothesis. The range of intensity is much larger (∼75% for Cy3 and ∼95% for Cy5) for this method of labeling, indicating that both Cy3 and Cy5 are very sensitive to the base pattern and therefore interact closely with the oligonucleotides.

For the Cy3 labeling, the consensus sequences are completely different from the case of direct labeling, with multiple dTs in all highly fluorescent sequences and multiple dAs in all poorly fluorescent sequences (see Table 2). A nucleoside pattern shift is not unexpected since the dyes should have reduced ability to form direct stacking interactions with the oligonucleotides, but a reversal of purines and pyrimdines indicates a completely different mode of interaction. Cy-dye protein labels have charged sulfonate groups on the aromatic rings to improve water solubility [45], [46]. This modification may also reduce their ability to associate with DNA via stacking interactions. The nucleoside monophosphate data of Harvey et al [23] includes the relative fluorescence yield of versions of Cy3 with and without charged sulfonate groups and indicates that the sulfonates do weaken the interaction with DNA, but the nucleoside-specific relative quantum yields remain similar: dG≈dA>dT≈dC.

**Table 2:** Normalized relative fluorescence intensity of the ten brightest and darkest 5-mers with 5′-biotin and labeled with streptavidin-Cy3 or -Cy5.

Rank	biotin-streptavidin Cy3-sequence	Normalized fluorescence†	biotin-streptavidin Cy5-sequence	Normalized fluorescence†
1	TTTTT	1.000±0.027	GTCAA	1.000±0.041
2	TTCTT	0.943±0.031	GTGAA	0.952±0.043
3	TTTTC	0.941±0.026	GGAAA	0.948±0.042
4	TTTTG	0.939±0.044	GGTAA	0.943±0.041
5	TTTCT	0.901±0.028	GGCAA	0.940±0.040
6	TTTGG	0.886±0.028	GCCAA	0.929±0.038
7	TTTCA	0.874±0.035	GTTAA	0.920±0.035
8	TTCTG	0.861±0.026	GAGAA	0.917±0.044
9	TCTTT	0.860±0.028	GACAA	0.911±0.040
10	TTCTC	0.858±0.030	GTAAA	0.908±0.044
1015	AACGA	0.398±0.021	AAGCG	0.093±0.019
1016	AAATA	0.394±0.026	AACCC	0.093±0.018
1017	AAACT	0.382±0.018	ACAGC	0.092±0.018
1018	CAAAA	0.381±0.019	AATTC	0.088±0.019
1019	CAGAC	0.380±0.025	AAACT	0.086±0.018
1020	AAACA	0.357±0.016	CATTC	0.086±0.020
1021	AAAAA	0.353±0.015	AACTC	0.073±0.019
1022	CAAAC	0.348±0.023	AAATC	0.072±0.018
1023	AAAAG	0.333±0.024	AACGT	0.068±0.021
1024	AAAAC	0.251±0.029	AAACC	0.061±0.020

† Error calculated as standard error of the mean.

In the case of labeling with Cy5-conjugated streptavidin, the consensus sequence for high fluorescence reverts back to the purine pattern with dG as the most favored base in the 5′-position and dAs favored elsewhere. However, the consensus sequence is distinctly different from those arising from direct labeling. Although a 5′-dG appears in all of the most highly fluorescent sequences, Cy5 appears to be interacting primarily with the two bases on the 3′-end of the experimental sequence. Looking at these two bases only, Cy5 requires a dA in both positions for strong fluorescence. This correlation is so strong that all of the possible 64 sequences with a pair of 3′-dAs are in the first 64 most fluorescence sequence positions, and the drop in relative fluorescence over this range of sequences is ∼50%. Low fluorescence is also correlated with dC and dT occupying the 3′-end. Looking only at the 5-mer homopolymer subset, the fluorescence intensity follows the same base pattern as with direct labeling with Cy5, dA>dG>dT>dC, which suggests a common interaction mechanism. The fact that the two bases on the 3′-end dominate the interaction may indicate that the Cy5 is positioned on the streptavidin in a way which prevents it from stacking at the end of the sequence, but rather positions the dye three bases further down, where it may stack between two bases. Table 3 summarizes intensity ranking of bases for all four labeling experiments.

**Table 3:** Nucleobase order based on relative fluorescence intensity for the 5-mer homopolymer sequence subset for each of the four labeling methods, as well as relative intensity and rank.

Label	Base order	Relative intensity	Rank (1–1024)
Cy3	G>A>T>C	1, 0.87, 0.75, 0.54	18, 243, 706, 1023
Cy5	A>G>T>C	1, 0.88, 0.61, 0.46	7, 49, 733, 1003
Cy3-streptavidin-biotin	T>G>C>A	1, 0.83, 0.58, 0.35	1, 18, 608, 1021
Cy5-streptavidin-biotin	A>G>T>C	1, 0.08, 0.07, 0.06	64, 540, 707, 934

Compared with direct labeling of oligonucleotides with Cy3/Cy5 phosphoramidites, labeling with dye conjugated streptavidin clearly leads to more complex modes of interaction. This is likely due to the additional complexity and variability introduced with this mode of labeling. Streptavidin is a tetrameric protein with each of its four units capable of binding biotin. This allows for the possibility that each streptavidin is binding one to four oligonucleotides. In addition, the dye is conjugated via a succinimidyl ester group which can react with non-protonated primary and secondary aliphatic amines on the protein. This results in attachment points on the protein which may be variable in both location and in number, and may depend on the reaction conditions. Gruber et al. [47], examined the fluorescence of Cy3 and Cy5 protein labels and found significant intensity differences between the dyes as well as differences resulting from the attachment method and density. Among their results, they found that the intensity of Cy3, but not Cy5, was enhanced by attachment to proteins and that Cy5 is more susceptible to quenching in multiply-labeled proteins. The complexity introduced by indirect labeling makes interpretation of the sequence-dependent results uncertain, but our results clearly show that this labeling method should be avoided in favor of direct labeling via phosphoramidites, which result in surprisingly large, but still lesser sequence-dependent modulation of fluorescence intensity.

In summary, common techniques based on fluorescent labeling of ssDNA are subject to a significant sequence-dependent bias. Sequence-dependent fluorescence intensity, combined with fluorescence quenching resulting from surface dye-dye interactions [48], may result in reduced linearity in microarray applications. While our results are directly applicable only to end-labeling with Cy3 and Cy5, gene-specific dye biases often found in microarrays using deoxynucleoside triphosphate (dNTP) internal labeling, as well as other dyes, suggest sequence-dependent variability, but of unknown magnitude. Cy3/Cy5 dye swaps, at least in experiments using end-labeling, should not significantly mitigate dye bias because the fluorescence of both of these dyes exhibits similar base-specific sequence dependence. cRNA labeling with Cy3 and Cy5 is likely to be subject to similar sequence-dependent bias due to the similarities between RNA and DNA. RT-PCR assays using the same cyanine dyes cannot be used to independently validate microarray data since the oligonucleotide or residual oligonucleotide fragment on the reporter dye may subject the results to a similar sequence bias. RT-PCR, as well as FISH, may benefit from the introduction of specific fixed-base sequences between the reporter dye and the design sequences to limit sequence-dependent variability and to increase the fluorescence signal. High-throughput DNA sequencing-by-synthesis should be particularly sensitive to sequence-dependent dye bases because almost all combinations of short base sequences will be repeatedly encountered, and detection failures (deletion errors) from sequences highly unfavorable to fluorescence would be systematic. Furthermore, the optical systems of sequencers need to carefully balance dynamic range of detection with throughput rate, making them vulnerable to dyes with significant variations in fluorescence [49]. Microarray-based detection of dye biases is a precise and high-throughput method, and may prove useful in evaluating other commonly used fluorescent labels as well as for assessing the suitability of new dyes.

## Supporting Information

Figure S1 **Cy3 5′-endlabeled DNA 5-mers, most fluorescent half, most intense to least intense.**
(TIF)

Figure S2 **Cy3 5′-endlabeled DNA 5-mers, least fluorescent half, most intense to least intense.**
(TIF)

Figure S3 **Cy5 5′-endlabeled DNA 5-mers, most fluorescent half, most intense to least intense.**
(TIF)

Figure S4 **Cy5 5′-endlabeled DNA 5-mers, least fluorescent half, most intense to least intense.**
(TIF)

Figure S5 **Cy3-streptavidin-biotin 5′-endlabeled DNA 5-mers, most fluorescent half, most intense to least intense.**
(TIF)

Figure S6 **Cy3-streptavidin-biotin 5′-endlabeled DNA 5-mers, least fluorescent half, most intense to least intense.**
(TIF)

Figure S7 **Cy5-streptavidin-biotin 5′-endlabeled DNA 5-mers, most fluorescent half, most intense to least intense.**
(TIF)

Figure S8 **Cy5-streptavidin-biotin 5′-endlabeled DNA 5-mers, least fluorescent half, most intense to least intense.**
(TIF)

Data S1 **Fluorescence intensity data for all labeling methods in spreadsheet format.**
(XLS)

Table S1 **Chemical synthesis protocols for dT coupling (representative of all four bases) and for the terminal label coupling.**
(PDF)
