# Supplementary figures and images for: Sequence-Dependent Fluorescence of Cyanine Dyes on Microarrays
Source: PLoS One. 2011 Jul 25;6(7):e22177. doi: 10.1371/journal.pone.0022177 (PMC3143128; doi:10.1371/journal.pone.0022177)

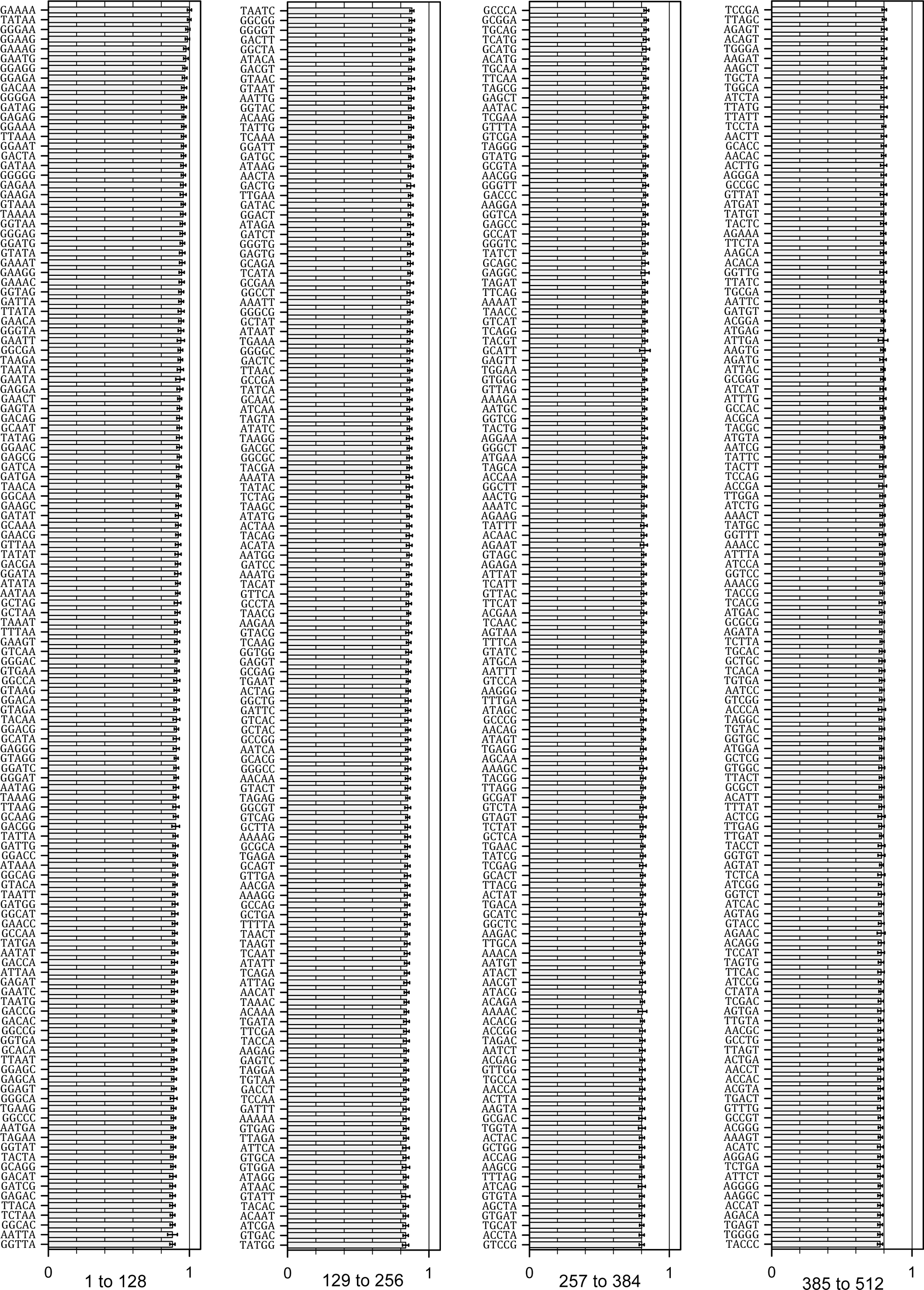

Supplement: Figure S1 — Cy3 5′-endlabeled DNA 5-mers, most fluorescent half, most intense to least intense. (TIF) [file pone.0022177.s001.tif]

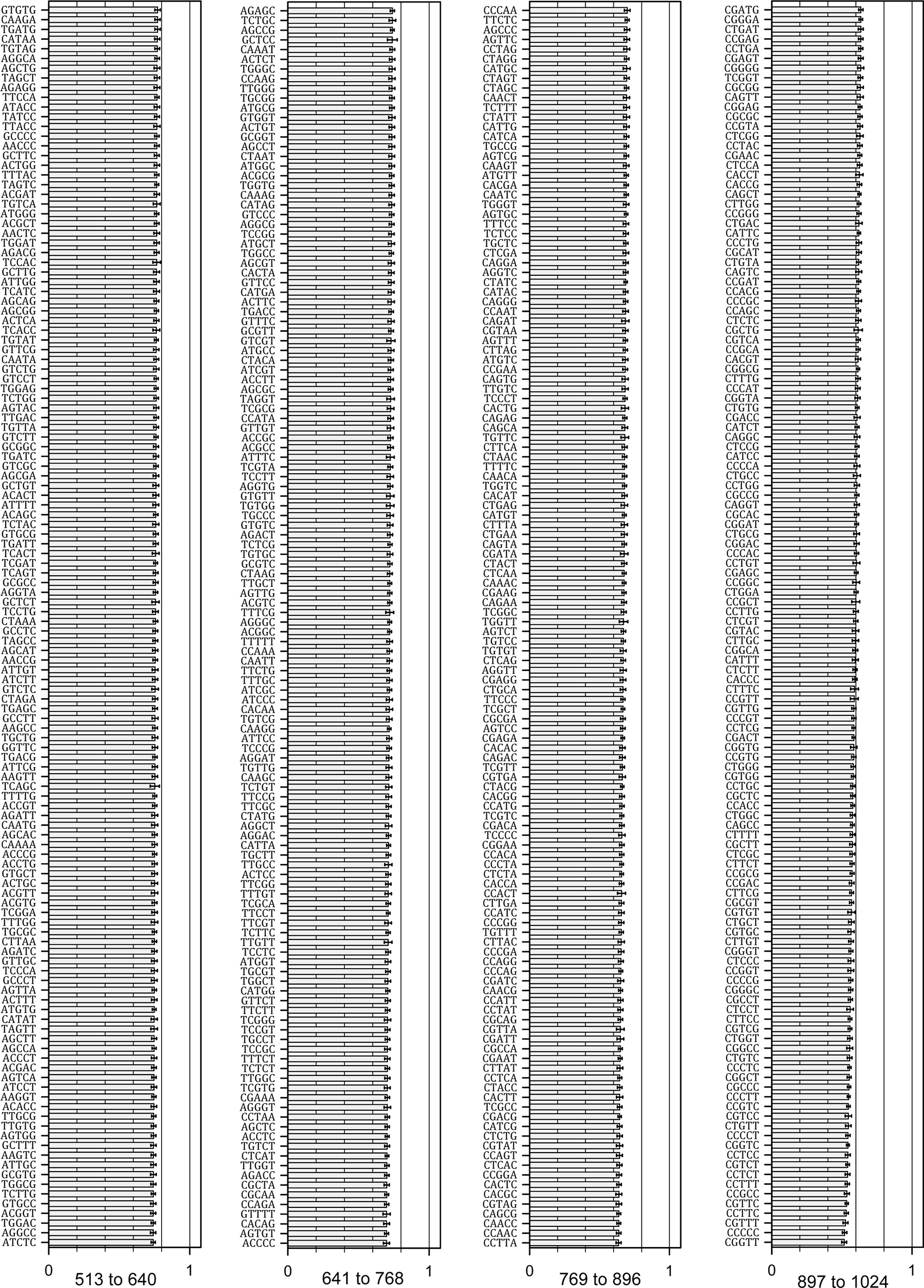

Supplement: Figure S2 — Cy3 5′-endlabeled DNA 5-mers, least fluorescent half, most intense to least intense. (TIF) [file pone.0022177.s002.tif]

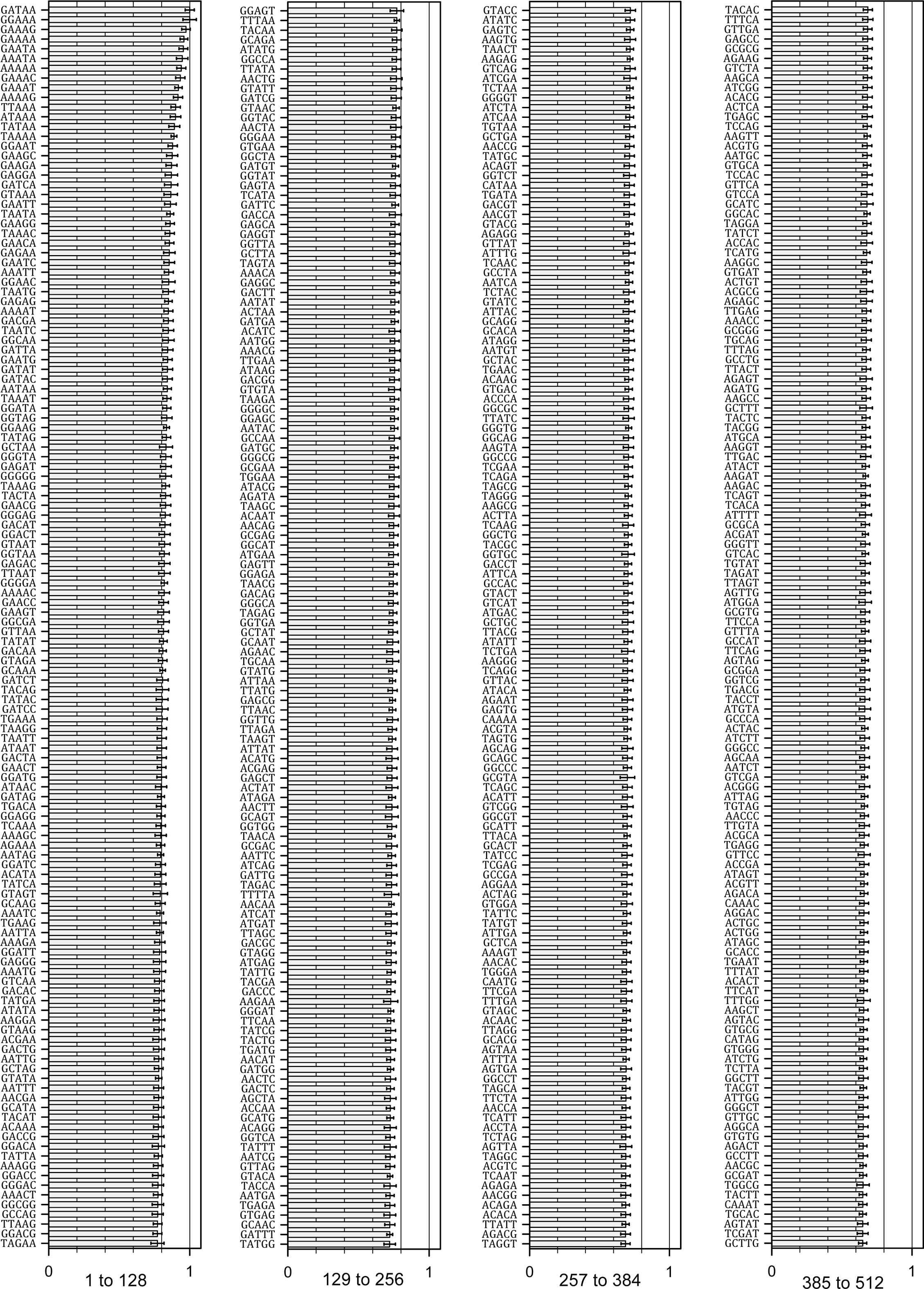

Supplement: Figure S3 — Cy5 5′-endlabeled DNA 5-mers, most fluorescent half, most intense to least intense. (TIF) [file pone.0022177.s003.tif]

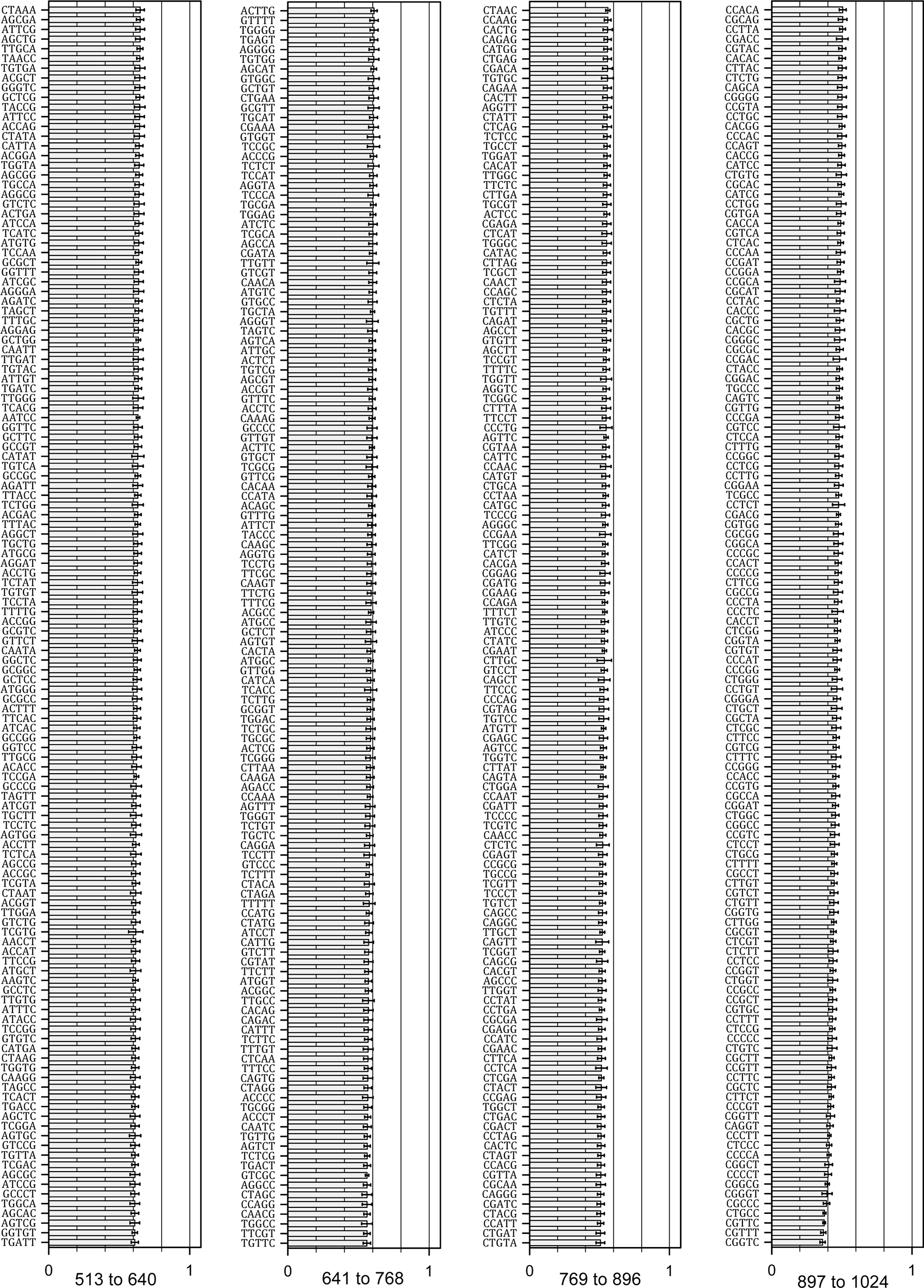

Supplement: Figure S4 — Cy5 5′-endlabeled DNA 5-mers, least fluorescent half, most intense to least intense. (TIF) [file pone.0022177.s004.tif]

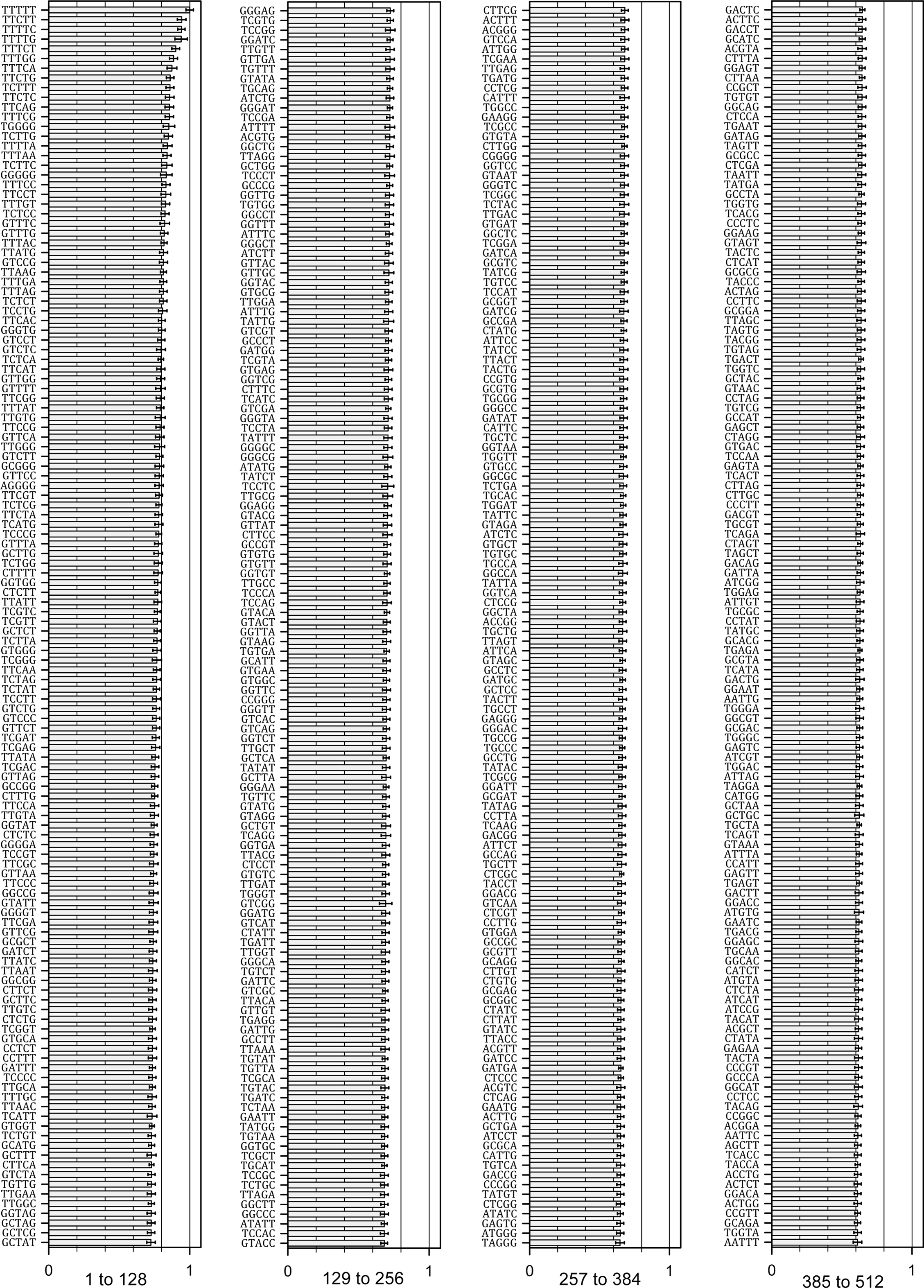

Supplement: Figure S5 — Cy3-streptavidin-biotin 5′-endlabeled DNA 5-mers, most fluorescent half, most intense to least intense. (TIF) [file pone.0022177.s005.tif]

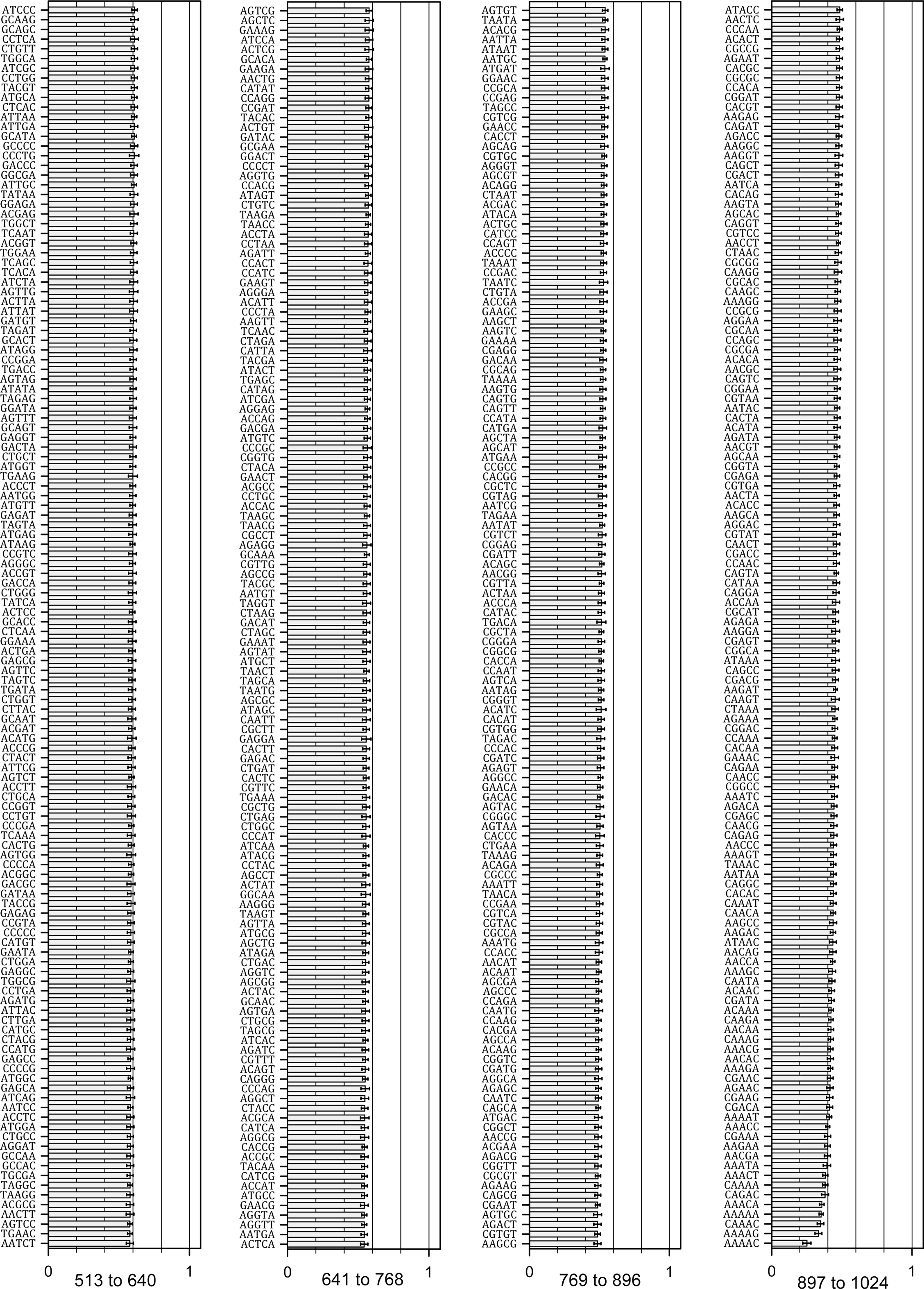

Supplement: Figure S6 — Cy3-streptavidin-biotin 5′-endlabeled DNA 5-mers, least fluorescent half, most intense to least intense. (TIF) [file pone.0022177.s006.tif]

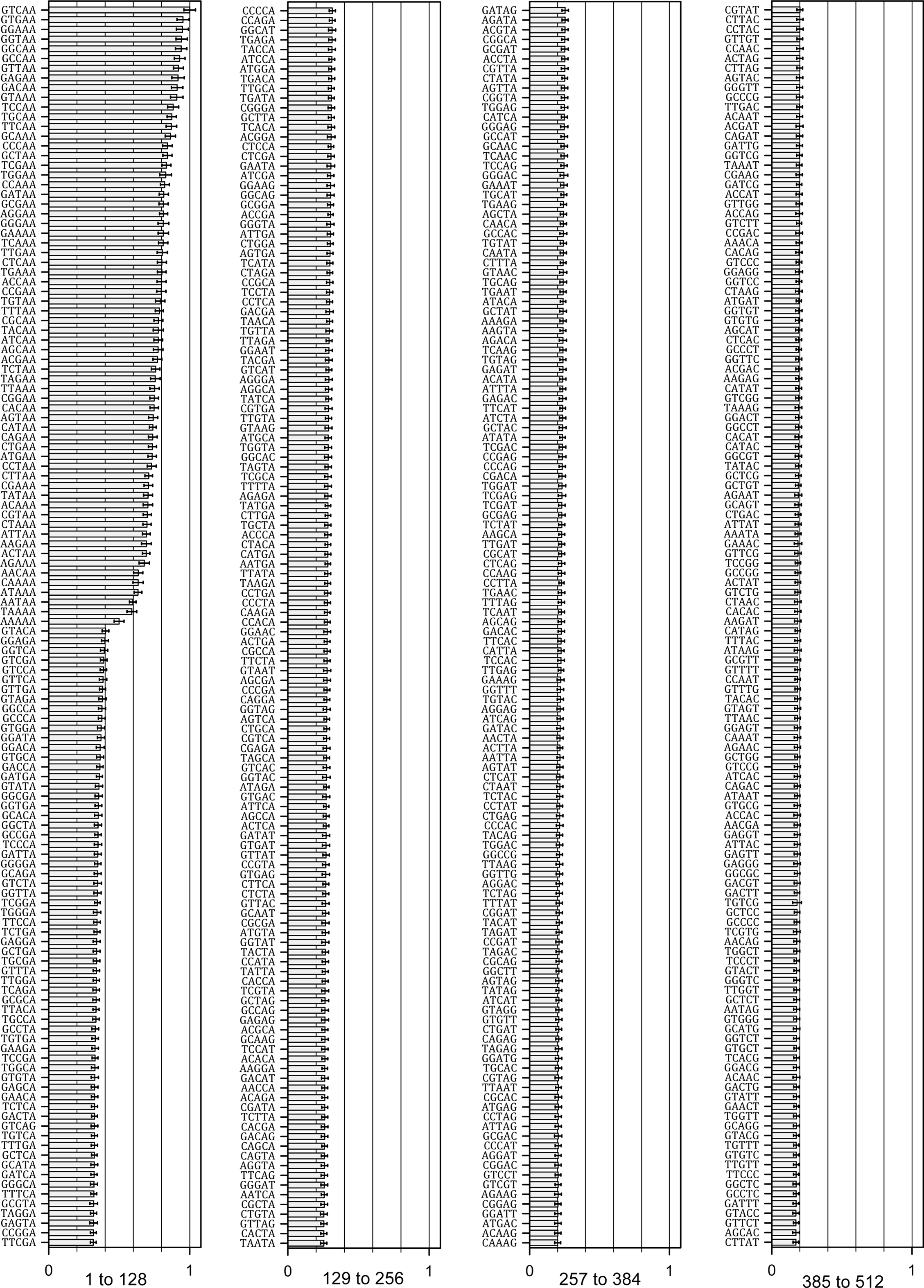

Supplement: Figure S7 — Cy5-streptavidin-biotin 5′-endlabeled DNA 5-mers, most fluorescent half, most intense to least intense. (TIF) [file pone.0022177.s007.tif]

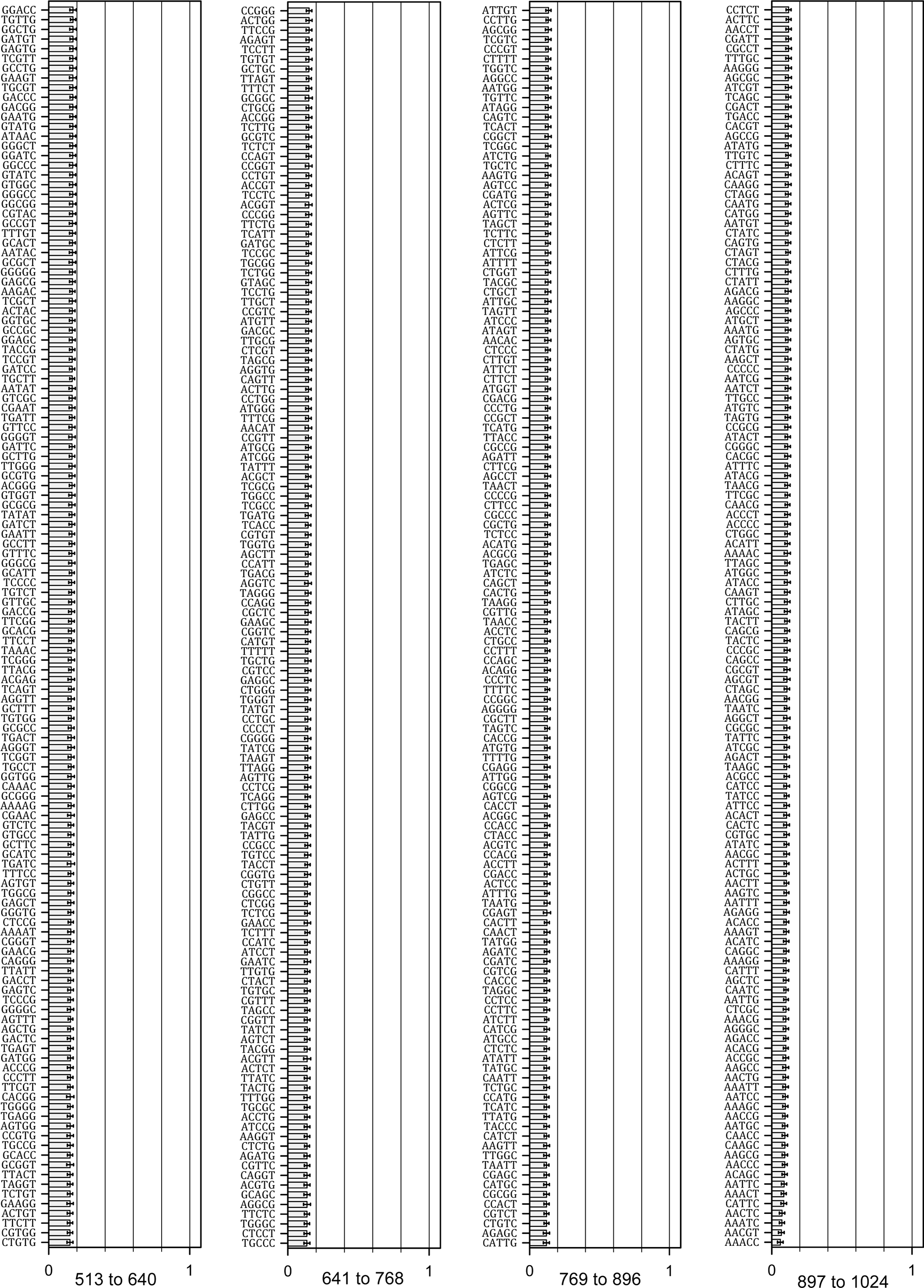

Supplement: Figure S8 — Cy5-streptavidin-biotin 5′-endlabeled DNA 5-mers, least fluorescent half, most intense to least intense. (TIF) [file pone.0022177.s008.tif]
