# Supplementary material for: Sequence-Dependent Fluorescence of Cyanine Dyes on Microarrays
Source: PLoS One. 2011 Jul 25;6(7):e22177. doi: 10.1371/journal.pone.0022177 (PMC3143128; doi:10.1371/journal.pone.0022177)
Supplement: Table S1 — Chemical synthesis protocols for dT coupling (representative of all four bases) and for the terminal label coupling. (PDF) [file pone.0022177.s010.pdf]

Cycle T

Function	Mode	pulse	sec	Description
**$Coupling**				
1 /*Wsh	*/ PULSE	20	0	"Flush system with Wsh"
2 /*Act	*/ PULSE	6	0	"Act"
21 /*T + Act	*/ PULSE	5	0	"T + Act"
2 /*Act	*/ PULSE	8	0	"Chase with Act"
1 /*Wsh	*/ PULSE	3	60	"Couple monomer"
1 /*Wsh	*/ PULSE	10	0	"Flush system with Wsh"
**$Capping**				
13 /*Caps	*/ PULSE	10	0	"Caps"
13 /*Caps	*/ PULSE	10	30	"Caps"
12 /*Wsh A	*/ PULSE	30	0	"Wsh A"
40 /*Gas A	*/ PULSE	1	30	"Dry column"
**$Oxidizing**				
12 /*Wsh A	*/ PULSE	45	0	"Flush system with Wsh A"
17 /*Aux	*/ PULSE	45	0	"Aux"
130 /*Event 2 Out	*/ NA	4	3	"Event 2 Out"
17 /*Aux	*/ PULSE	16	60	"Aux"
12 /*Wsh A	*/ PULSE	25	0	"Flush system with Wsh A"

Cycle M (Cy3/Cy5/biotin)

Function	Mode	pulses	sec	Description
**$Coupling**				
1 /*Wsh	*/ PULSE	20	0	"Flush system with Wsh"
2 /*Act	*/ PULSE	6	0	"Act"
25 /*8 + Act	*/ PULSE	5	0	"8 + Act"
2 /*Act	*/ PULSE	8	0	"Chase with Act"
1 /*Wsh	*/ PULSE	3	300	"Couple monomer"
1 /*Wsh	*/ PULSE	20	0	"Flush system with Wsh"
1 /*Wsh	*/ PULSE	10	0	"Flush system with Wsh"
2 /*Act	*/ PULSE	6	0	"Act"
25 /*8 + Act	*/ PULSE	5	0	"8 + Act"
2 /*Act	*/ PULSE	8	0	"Chase with Act"
1 /*Wsh	*/ PULSE	3	300	"Couple monomer"
1 /*Wsh	*/ PULSE	10	0	"Flush system with Wsh"
**$Oxidizing**				
15 /*Ox	*/ PULSE	30	0	"Oxidize"
12 /*Wsh A	*/ PULSE	300	0	"Wsh A"
12 /*Wsh A	*/ PULSE	300	300	"Wsh A"
